# Highly Sensitive Hybrid Nanostructures for Dimethyl Methyl Phosphonate Detection

**DOI:** 10.3390/mi12060648

**Published:** 2021-05-31

**Authors:** Sanjeeb Lama, Jinuk Kim, Sivalingam Ramesh, Young-Jun Lee, Jihyun Kim, Joo-Hyung Kim

**Affiliations:** 1 INHA IST and Laboratory of Intelligent Devices and Thermal Control, Department of Mechanical Engineering, Inha University, Incheon 22212, Korea; sanjeeblama132@gmail.com (S.L.); juzzang95@naver.com (J.K.); jihyunn.kim@inha.ac.kr (J.K.); 2 Department of Mechanical, Robotics and Energy Engineering, Dongguk University-Seoul, Seoul 04620, Korea; ramesh74@naver.com

**Keywords:** chemical warfare agent (CWAs), dimethyl methylphosphonate (DMMP), volatile compounds (VOCs), quartz crystal microbalance (QCM), surface acoustic wave (SAW)

## Abstract

Nanostructured materials synthesized by the hydrothermal and thermal reduction process were tested to detect the dimethyl methylphosphonate (DMMP) as a simulant for chemical warfare agents. Manganese oxide nitrogen-doped graphene oxide with polypyrrole (MnO_2_@NGO/PPy) exhibited the sensitivity of 51 Hz for 25 ppm of DMMP and showed the selectivity of 1.26 Hz/ppm. Nitrogen-doped multi-walled carbon nanotube (N-MWCNT) demonstrated good linearity with a correlation coefficient of 0.997. A comparison between a surface acoustic wave and quartz crystal microbalance sensor exhibited more than 100-times higher sensitivity of SAW sensor than QCM sensor.

## 1. Introduction

**Figure 1:**
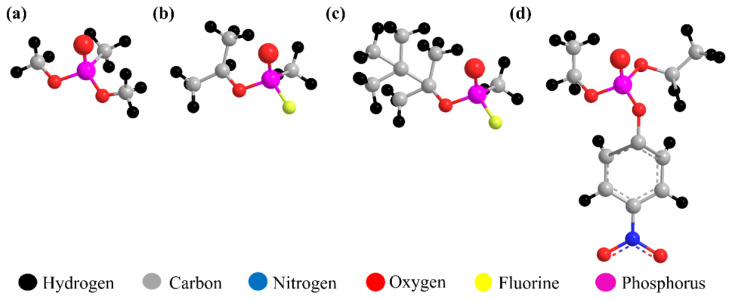
Chemical structure of a simulant and nerve agents: (**a**) DMMP, (**b**) sarin, (**c**) soman, and (**d**) paraoxon.

Lethal CWAs even at a lower dosage should be rapidly detected because of their toxic nature, rapid action, and irreversibility [1,2]. For instance, sarin (GB) is a colorless and odorless compound containing a highly toxic phosphonate structure that acts as an inhibitor of the enzyme acetylcholinesterase and distorts neuromuscular transmission [3]. The traditional detecting methods—gas chromatography, liquid chromatography, ion mobility spectroscopy, atomic emission detection, Fourier transform infrared (FT-IR), and Raman spectroscopy—possess high sensitivity, reliability, and precision, however they require expensive equipment, highly skilled operators, as well as time-consuming analytical processes for field applications. In order to satisfy on-site monitoring of CWAs, devices with a low power consumption and a low-cost with portability are highly preferable [4,5]. Several hand-held type sensors have been suggested and studied which are, SAW sensor [6,7], QCM sensor [8,9], chemi-resistive type sensor [10,11], and cantilever-based membrane sensor [12,13]. Based on these sensors, to detect the target CWA molecules, sensing materials are applied. Due to the regulation of the original agents, nontoxic and organophosphorus DMMP is used as a simulant for CWAs, such as the nerve agents of G-series, sarin, soman (GD), and paraoxon [14] as shown in Figure 1.

QCM sensors are widely used in various fields due to their compatibility with different operating conditions in the gaseous/liquid medium. This fashion has been coordinated by the parallel development in tailored electronic interfacing systems for QCM based sensors. Several QCM electronic measurements systems exist in the literature, changing in their level of complexity, cost, accuracy, and portability. These interfacing circuits aim to precisely measure physical variations that are related to relevant physical quantities.

Abad et al. reported that the piezoelectric transducer uses QCM to sense the signal produced by enzymes [15]. The enzyme was anchored on the face of the quartz crystal. When the interaction between the substrate and enzyme occurred, it generated a change in the resonant frequency of the crystal; thus, the mass or the surface property of the crystal was verified in real time. Nivens et al. reported the effect produced by the adhesion of microbial deposits to a crystal vibration frequency deposited on QCM crystal’s face [16]. The process is mainly used in food and beverage industries by employing biofilms. There are many commercial versions of the QCM device widely used to detect the initial adhesion of bacteria to stainless steel surfaces. This technique has proven to be efficient for the identification of bacteria adhesion. However, QCM sensors suffers from two main disadvantages: 1. The distribution of sensitivity profile and displacement of conventional QCM are influenced by the electrode structure which results in non-uniform sensitivity, and finally, degradation of its performance [17], and 2. Low resonant frequency inhibits the performance of the QCM as the conventional QCM are designed to operate at 5–10 MHz [18].

To detect DMMP as a simulant, several promising candidates such as polyhedral oligomeric silsesquioxane (POSS) [8] and thiourea (TU) [19] showed good sensitivity. Carbon nanotubes with tubular nanostructures, since their discovery by Iijima in 1991, have been studied extensively due to their excellent performance for many applications such as gas sensing [20,21], gas storage, energy conversion, catalysis, optoelectronics, and drug release. N-MWCNTs have been demonstrated by successful use for oxygen reduction reactions as well as the ability of similar carbon–nitrogen structures to catalytically split water in the production of hydrogen [22,23]. Another approach using graphene oxide as a sensitive layer in the detection of DMMP was reported [24]. The incorporation of copper phthalocyanine in conducting polypyrrole (PPy) electrochemically was suggested to detect DMMP, nerve agents, and VOCs [25]. It was reported that the response behavior of single-walled carbon nanotube (SWNT) with polyaniline exhibited a sharp response, good reproducibility, and linearity at room temperature concentration [26]. In the gas phase detection, the decomposition of DMMP over manganese oxide catalysts in the presence of light was observed as a strong adsorption with physisorption and chemisorption [27].

This paper presents the experimental study of various nanostructured composite materials for-sensitivity, linearity, selectivity, reproducibility, and response/recovery times to detect DMMP, VOCs, and water. For comparison, SAW and QCM sensors were tested under same conditions. The effect of relative humidity on the sensitivity of the QCM sensor was also investigated.

## 2. Materials and Methods

### 2.1. Fabrication of Composite Sensing Materials

**Figure 2:**
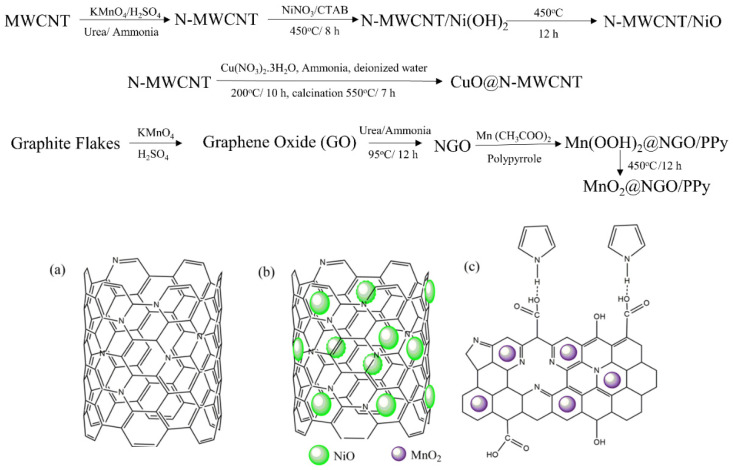
Synthesis processes and structure of (**a**) N-MWCNT, (**b**) N-MWCNT@NiO, and (**c**) MnO_2_@NGO/PPy.

Based on our early approach for sensing materials for CWAs, hybrid nanostructured composite materials were synthesized by the hydrothermal and thermal reduction process. The efficient hydrothermal chemical process was adopted in order to prepare nitrogen-doped graphene oxide (NGO) and facile synthesis of NGO with MnO_2_ decorated with conducting polymer, pyrrole [28]. N-MWCNT and N-MWCNT@NiO were synthesized by the thermal reduction chemical reaction followed by calcination at high temperature as reported in our previous study [29]. The N-MWCNT decorated by CuO which resembles the nanosheet shape structure was prepared by a hydrothermal process in the presence of urea [30]. Figure 2 represents the synthesis processes and chemical structure of N-MWCNT, N-MWCNT@NiO, and MnO_2_@NGO/PPy.

**Figure 3:**
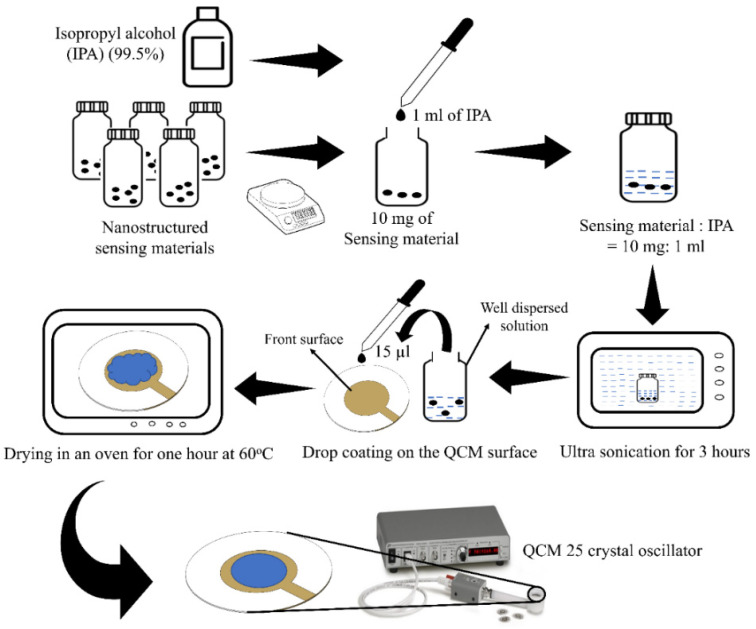
Thin sensing layer deposition by drop coating process.

The synthesized composite materials were mixed with isopropyl alcohol (IPA) in the ratio of 10 mg:1 mL. The mixture was ultrasonicated for 3 h followed by 15 µL drop coating on the front surface of the QCM sensor. The coated QCM sensors were dried in an oven for one hour at 60 °C. The sensors were cooled at room temperature and used in the QCM oscillator without further processing. The thin film sensing layer on the QCM was formed by the drop coating process as shown in Figure 3. A 1-inch diameter size thin membrane type QCM sensor (Stanford Research System, SRS) has a circular Cr/Au electrode on both sides, operating at a resonant frequency of 5 MHz [31].

### 2.2. Characterization Apparatus and Selectivity Conditions

VOCs—ethanol, toluene, n-hexane, and methanol—and water were tested because these compounds can act as potential interferences during the detection of DMMP on earth. VOCs were achieved respectively; ethanol (95%) from Merck, Darmstadt, Germany, toluene (99.5%) from Duksan, Ansan, Korea, n-hexane (95%) from Avantor, Radnor, PE, USA, methanol (99.80%) and Isopropyl alcohol (IPA) (99.5%) from Daejung, Busan, Korea.

DMMP (97%, Sigma Aldrich, St. Louis, MO, USA) is a colorless liquid having a molecular weight of 124.08 g/mol with a density of 1.145 g/mL at 25 °C. It is reported that a boiling and melting point of DMMP at 181 °C and −50 °C, respectively.

**Figure 4:**
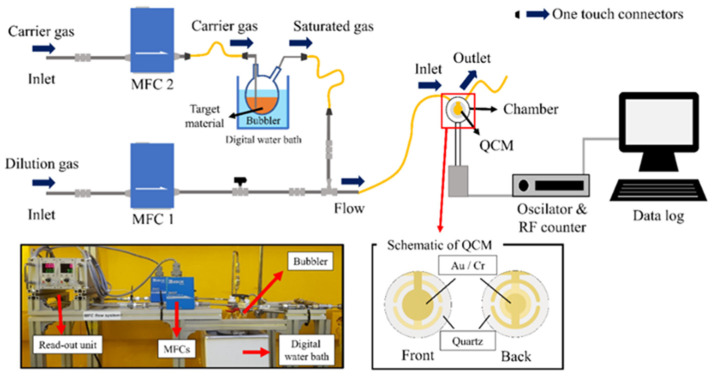
Schematic diagram of the gas sensing system.

The gas feeding system was designed to test the different concentrations of the saturated vapors of DMMP and VOCs detection with a QCM sensor, which is illustrated in Figure 4. The system consists of two mass flow controllers (MFCs, KOFLOC, Nagoya, Japan) connected to one-way valves, steel pipes for gas feeding, a vapor bubbler system, and one-touch connectors.

If the QCM detects the vapors of DMMP or VOCs in the feeding gas, the frequency change due to the amount of adsorption of the target vapors on the sensing layer can be monitored by a QCM 200 digital controller (SRS). A general-purpose interface bus (GPIB interface) was used to transfer the raw data from the digital controller to the computer.

The testing chamber allows an axial flow of the vapors flowing radially outward from the input port located at the center of the chamber to the exit at the edge of the chamber, in a volume of 150 µL providing with the highest sensitivity by overlapping the area of the flat QCM oscillator [31]. The saturated vapors are generated by the flow of the carrier gas through the inlet of the bubbler. Then nitrogen acts as the carrier as well as dilution gas.

Then, the output flow rate of DMMP vapor can be formulated using a bubbler equation from [32]:

(1) $$F_{DMMP}=\left(\frac{P_{DMMP}\times F_{c}}{P_{0}-P_{DMMP}\;}\right)$$

where *F_c_* is the flow rate of carrier gas from 48 to 320 sccm (standard cubic centimeters per minute), *P*_0_ is the outlet pressure in the bubbler and kept at 760 mmHg in these experiments, and *P_DMMP_* is the vapor pressure of the DMMP which can be calculated from the Antoine Equations (2) and (3) [19] expressed as below:

(2) $$ln\;P_{DMMP}=\left(A-\frac{B}{C+T}\right)$$

(3) $$log\;P_{DMMP}=\left(A-\frac{B}{C+T}\right)$$

where *T* is the temperature of the bubbler (Here, *T* is set as 298 K) and *A, B*, and *C* are the coefficients that are used in the Antoine equation for various vapors as given in Table 1.

The saturated and the dilution gas were fed to the gas line to blend in together homogeneously that regulates the adsorption of saturated vapors on the sensing adsorbent. The maximum flow rate was 2320 sccm, which is a mixture of saturated gases and dilution gas, while the minimum flow rate of 2000 sccm was purging with dilution gas only. The resultant DMMP concentration was calculated from the output flow rate and the dilution ratio [37]

(4) $$C_{DMMP}(\mathrm{ppm})=\left(\frac{\;F_{DMMP}\times 10^{6}}{F_{d}+F_{c}+F_{DMMP}}\right)$$

where *F_d_* is the flow rate of the dilution gas, which is at 2000 sccm in this study. After each test, the sensors were cleaned by a purging process with nitrogen to eliminate the attached DMMP from sensor surface. All the experiments were carried out at room temperature and 1 atm. For the sensing performance of gas sensors, some important characteristics such as sensitivity, selectivity, response/recovery speed, stability, and reproducibility, were tested [38].

## 3. Results

**Figure 5:**
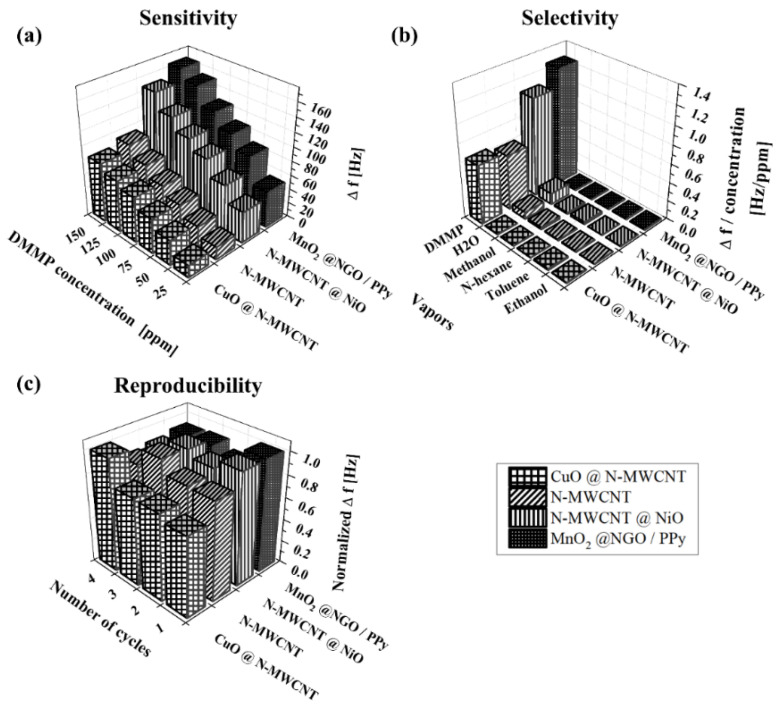
Comparison of (**a**) sensitivity (**b**) selectivity and (**c**) reproducibility of CuO@N-MWCNT, N-MWCNT, N-MWCNT@NiO, and MnO_2_@NGO/PPy.

It is natural that the frequency shift also increases when the QCM sensor was tested with an increment of the analyte concentration of DMMP vapor ranging from 25 to 150 ppm as shown in Figure 5a. From the experimental result for CWA sensing performance with synthesized nanocomposites on QCM, MnO_2_@NGO/PPy exhibited an excellent sensitivity where the individual compound has exceptional properties in the detection of DMMP-manganese oxide [27], NGO [24], and conducting polymer PPy [25]. It seems that the sensitivity is about two-times better than CuO@MWCNT and N-MWCNT, which show a similar response of sensitivity with a little variation in the frequency shift between them. Additionally, adding NiO to N-MWCNT enhanced the sensitive performance to detect DMMP vapor due to the oxygen reduction reactions [23]. Further, we found that a fast recovery of sensing by rapid desorption took place when the chamber was purged with nitrogen gas, resulting in the reduction of QCM oscillating frequency.

The sensors were tested against several VOCs including ethanol, methanol, toluene and n-hexane, and H_2_O, each at a fixed flow rate of 200 sccm, which may act as interferences. The selectivity of the QCM sensors coated with various materials in respect of DMMP has to be compared under different environmental conditions in order to gain an understanding of how much extent the VOCs as well as water vapor can hinder the adsorption between the sensing composites and DMMP vapor. We observed that all the composite materials seemed to be more sensitive towards DMMP vapor than the interferences as shown in Figure 5b. For the detection of DMMP, the excellent sensitivity of 1.26 and 1.07 Hz/ppm was observed in the sensing layer of MnO_2_@NGO/PPy and N-MWCNT@NiO respectively. The measured sensor response can be explained by the interaction of hydrogen bond between DMMP and graphene oxide of the nanocomposite [24] or nanotube [39]. Therefore, it is proven that all the composite materials have a potential to possess an excellent sensitivity to the target CWA vapor.

To check the reproducibility of sensing performances of composites, we exposed the sensors to the constant concentration of DMMP for several times. Figure 5c shows the reproducibility of the QCM sensors with sensing nanocomposites at DMMP concentration of 25 ppm. Table 2 presents the sensor response and the standard deviation when it was exposed to a constant DMMP vapor. The response curves show similar behaviors under the same exposure and the recovery. From the results, we cannot find any significant changes in the sensor response which indicates an excellent reproducibility of the sensing nanostructured materials [5,26].

**Figure 6:**
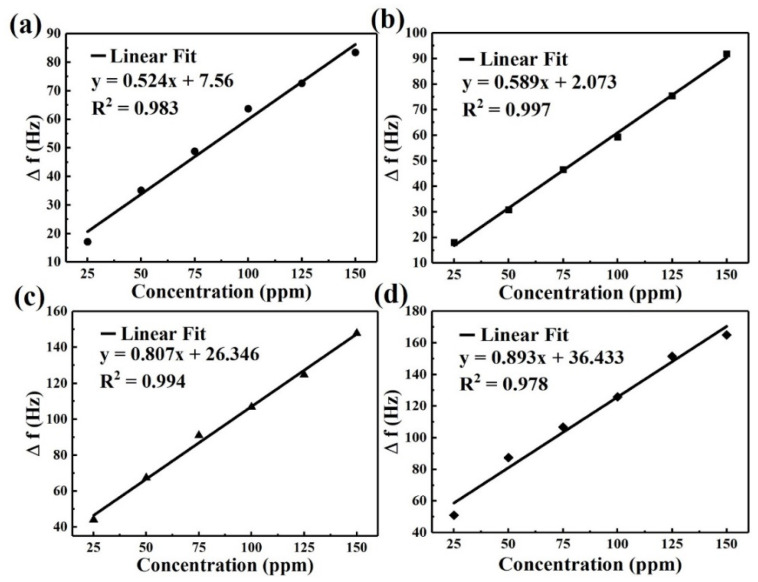
The linearity of frequency shift by (**a**) CuO@N-MWCNT, (**b**) N-MWCNT, (**c**) N-MWCNT@NiO, and (**d**) MnO_2_@NGO/PPy in the detection of different concentrations of DMMP.

Figure 6 shows the frequency shift as a function of the concentration of DMMP vapor in feeding gas. Note that the operating frequency linearly increases as DMMP vapor increases from 25 to 150 ppm. Immediate change in the frequency is observed with the absorption of DMMP when DMMP gas was moved into the chamber. N-MWCNT and N-MWCNT@NiO presents the highest correlation coefficient which may stem from the large surface area as well as a good adhesive behavior to electrode on sensor resulting in enhanced sensitivity response [4].

The response and recovery times are defined as the time interval to reach 90% of the equilibrium and return to 10% of the equilibrium baseline by purging with dry nitrogen respectively. Table 3 shows the response and recovery times of all synthesized nanocomposite under different concentrations of DMMP vapor. From the experimental results, the composite materials show rather longer recovery time than the response one. Here, the shorter response time indicates that it can react to the organophosphorus composition of the DMMP quickly and short recovery time depicts that the recovering ability of the sensing film to its equilibrium baseline value is swift.

As the concentration of DMMP increases, most of the nanocomposite materials shows a shorter response and recovery times relatively. The reversible response can imply that the adsorption of the analytes on the sensing film consists of weak bonds, such as hydrogen bonding or van der Waals force [40]. Table 4 illustrates the performance summary of the QCM sensors with different sensing materials used for detection of DMMP.

**Figure 7:**
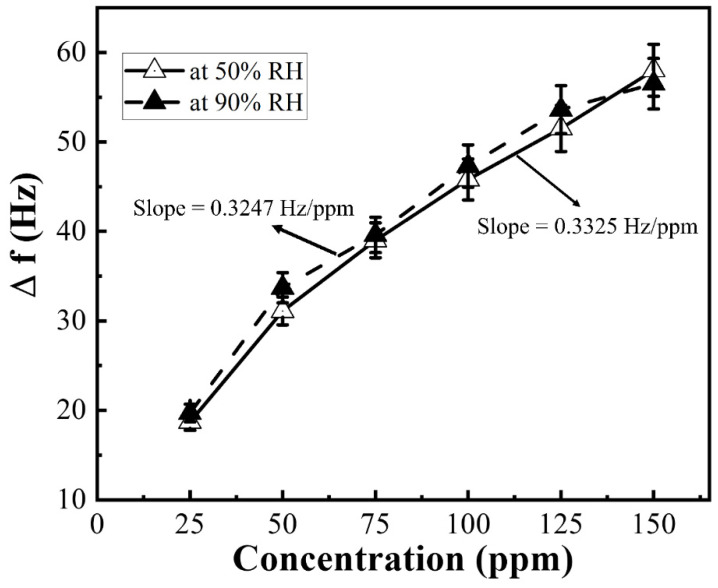
The sensitivity of N-MWCNT@NiO at 50 and 90% of relative humidity at 20 °C.

For the environmental effect on sensing performance, we also tested the response of the sensor under different relative humidity (RH) conditions. As N-MWCNT@NiO shows the highest sensitivity to water vapor in Figure 5b, we measured the frequency shift at the relative humidity of 50%, and 90% under a constant temperature of 20 °C with DMMP vapor ranging from 25 ppm to 150 ppm; the results are shown in Figure 7. The sensitivity of the sensor in 50% and 90% relative humidity at a constant temperature of 20 °C are 0.3325 ± 0.0255, and 0.3247 ± 0.0339 Hz/ppm, respectively. The decrement of sensitivity was due to the water molecules on the reacting surface [6]. When the relative humidity is higher than 90%, the sensor cannot work due to the absorption of thin water film on the QCM surface and fails to attain its basic frequency [48].

**Figure 8:**
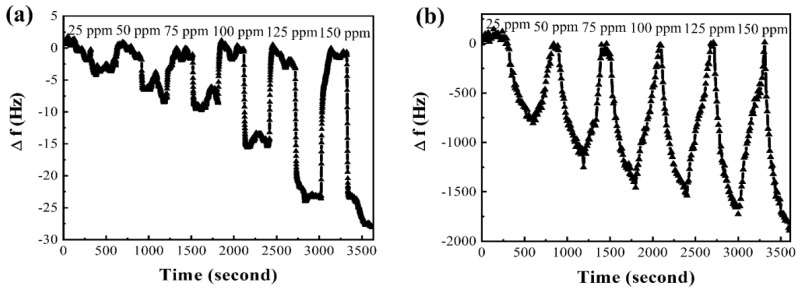
The sensitivity of a (**a**) QCM and (**b**) 250 MHz operating SAW sensor for different concentrations of DMMP at the same concentration of N-MWCNT@NiO.

Based on comparison of sensitivity, linearity, selectivity, reproducibility, and response/recovery times of the nanostructured composite materials, we selected a potential candidate to be tested by SAW sensor, designed by our previous studied. N-MWCNT@NiO in QCM and SAW sensors operating at 250 MHz were primarily selected and prepared. All the sensing performance were compared under the same concentrations of the solvent and DMMP vapor as shown in Figure 8. N-MWCNT@NiO was mixed with IPA at the ratio of 1 mg:4 mL because the SAW sensor is very sensitive of variation of mass change of coating layer, which can fail to produce good results when the weight of the coating materials is too heavy. From the Figure 8, the sensitivity in the SAW sensor is about 118 and 70-times higher than that of the QCM sensor at a DMMP concentration of 25 and 150 ppm, respectively, was observed.

## 4. Conclusions

We tested several novel hybrid nanostructured composite materials as a sensing material for the early detection of CWAs. Nanostructured composite materials were synthesized by the hydrothermal and thermal reduction process. The sensitivity, selectivity, linearity, reproducibility, and response/recovery times for different concentrations of DMMP vapor as a simulant and VOCs were measured and compared. Two different QCM and SAW sensors coated with N-MWCNT@NiO were compared in terms of sensitivity at a constant temperature of 20 °C. MnO_2_@NGO/PPy showed excellent sensitivity and selectivity for DMMP ranging from 25 to 150 ppm. All the nanostructured composite materials demonstrated excellent reproducibility for 25 ppm DMMP with no significant changes between four successive cycles of DMMP exposure and recovery. The response/recovery test exhibited a shorter response time and longer recovery compared with other synthesized materials. The RH test showed a decrement in the sensitivity of 7.8 × 10^−3^ Hz/ppm as the humidity increased from 50 to 90% RH. The comparison between a SAW and QCM sensor resulted in an increment of frequency shift by 118 and 70-times in the SAW sensor than in the QCM sensor at 25 and 150 ppm, respectively.

## Figures and Tables

**Table 1:** Coefficients used for various vapors in this study [33,34,35,36].

Vapors	Equation	Unit	A	B	C	Temp. Range [K]
DMMP	(2)	T (K), P (Pa)	22.31900	4340.00	−51.700	263.2–453.8
Ethanol	(3)	T (K), P (kPa)	7.24739	1599.04	−46.391	292.8–366.6
N-hexane	(3)	T (°C), P (mmHg)	6.87776	1171.53	224.366	286.18–342.7
Water	(3)	T (°C), P (mmHg)	8.07131	1730.63	233.426	274.0–373.0
Toluene	(3)	T (°C), P (mmHg)	6.95464	1344.80	219.482	279.0–409.0
Methanol	(3)	T (°C), P (mmHg)	8.07240	1574.99	238.870	257.0–364.0

**Table 2:** Sensor mean response and standard deviation.

Sensing Nanocomposite Film	Response (Hz)	Standard Deviation (Hz)
CuO@N-MWCNT	12.6	1.71
N-MWCNT	19.02	1.21
N-MWCNT@NiO	47.40	2.38
MnO_2_@NGO/PPy	47.29	2.69

**Table 3:** Response/recovery results of hybrid nanocomposites.

Sensor Characteristics		DMMP Concentration (ppm)				
	Nanocomposites	25	50	75	100	125
Response time (s) τ_90%_	CuO@N-MWCNT	~121	~115	~108	~104	~102
	N-MWCNT	~130	~100	~100	~101	~100
	N-MWCNT@NiO	~117	~104	~106	~103	~96
	MnO_2_@NGO/PPy	~136	~101	~103	~99	~123
Recovery time (s) τ_90%_	CuO@N-MWCNT	~155	~195	~113	~139	~143
	N-MWCNT	~193	~142	~135	~132	~170
	N-MWCNT@NiO	~206	~142	~150	~139	~144
	MnO_2_@NGO/PPy	~142	~123	~117	~118	~113

**Table 4:** The summary of the QCM sensors for detection of DMMP using various sensing materials.

Ref.	Materials	Concentration (ppm)	Response (Hz)	Response Time (s)	Recovery Times (s)
[41]	HFIPP-GR	5	71 ± 4	*T*_80_ < 108	*T*_80_ = 600
[42]	V_2_O_5_ coated ZnO nanorods	15	~40	*T*_80_ < 300	*T*_80_ > 900
[43]	Co_3_O_4_@gold/MWCNT/polypyrrole	60	90	*T*_98_ = 60	*T*_98_ = 493
[44]	In_2_O_3_-Au	50	<80	<100	~200
[45]	Zeolite Socony Mobil-5	20	~55	*T*_80_ < 100	-
[46]	WO_3_ nanoflake	3.91	<160	30	73
[47]	Polyvinylidene fluoride	150	~50	~60	~60
This work	MnO_2_@NGO/PPy	50	87	*T*_90_ = 101	*T*_90_ = 123
